# CO_2_-Responsive Wormlike Micelles Based on Pseudo-Tetrameric Surfactant

**DOI:** 10.3390/molecules27227922

**Published:** 2022-11-16

**Authors:** Xia Wei, Xiran He, Dongmei Zhang, Xin Su

**Affiliations:** 1 State Key Laboratory of Polymer Materials Engineering, Polymer Research Institute, Sichuan University, Chengdu 610065, China; 2 Research Institute of Experiment and Detection, Xinjiang Oilfield Company, Karamay 834000, China

**Keywords:** CO_2_ switching, tetrameric surfactant, viscosity, wormlike micelles, cyclen

## Abstract

Wormlike micelles, which are linear aggregates created by the self-assembly of surfactants, may entangle to form dynamic three-dimensional network-like structures, endowing solutions with considerable macroscopic viscoelasticity. Recently, a pressing need has arisen to research a novel stimuli-responsive worm-like micelle that is efficient and environmentally friendly. CO_2_ is an inexpensive, abundant, non-toxic, biocompatible, and non-combustible gas, and it is anticipated that CO_2_ may serve as the trigger for stimuli-responsive worm-like micelles. In this paper, the formation of CO_2_-switchable pseudo-tetrameric surfactants, which subsequently self-assemble into CO_2_-switched wormlike micelles, is accomplished using a simple mixing of two commercial reagents, such as stearic acids and cyclen. The rheological characteristics switched by the use of CO_2_ are cycled between that of a low-viscosity (1.2 mPa·s) fluid and a viscoelastic fluid (worm-like micelles, 3000 mPa·s). This article expands the field of study on stimuli-responsive worm-like micelles.

## 1. Introduction

Recent years have seen the emergence of wormlike micelles [1] as a significant study path in colloids and soft matter. A wormlike micelle is an equilibrium polymer or living polymer model with static and dynamic properties that differ from those of polymers for both long and short periods relative to its period of existence [2,3,4]. Wormlike micelles are candidates to replace polymers as viscosity enhancers and can be widely used in personal cleaning and care [4], drag-reducing fluid [5,6], solid particle transport [7], and fracturing fluids [5,8], among other industrial applications. A great deal of attention has been focused on the peculiar rheological features of wormlike micelles, which exhibit viscoelasticity like polymers but are not precisely comparable to polymers.

Wormlike micelles are also known as threadlike micelles [7,8,9] or giant micelles [10]. Under certain conditions, rodlike micelles of surfactant continue to grow in one dimension along the non-axial direction to form long flexible columnar micelles [1,2]. These micelles typically have diameters of a few nanometers, and persistent lengths of tens to hundreds of nanometers, while the contour length may reach several micrometers [2], producing micelles resembling worms. Under suitable conditions with proper temperature, concentration, and counterion, wormlike micelles interweave and form a temporary three-dimensional transient network, therefore imparting to the system a macroscopic viscoelasticity comparable to that of polymer solutions. However, wormlike micelles are held together by weak intermolecular non-covalent bonds (bond energy, 40 kJ/mol) [11], whereas polymers are held together by strong covalent bonds (bond energy, 100–900 kJ/mol) [12] between monomers with fixed molecular weight and molecular weight distribution, and their rheological properties are typically shear irreversible, i.e., the solution viscosity cannot be recovered after their molecular chains are broken at high shear rates. At the microscopic level, it is a dynamic equilibrium system, and the size of the wormlike micelles changes in response to temperature, concentration, and type of components. This dynamic equilibrium system imparts shear-reversible viscoelasticity; hence, worm-like micelles are sometimes known as living polymers [1,2], or equilibrium polymers [13,14,15].

Because of their unusual microstructure and modifiable rheological characteristics, stimuli-responsive wormlike micelles have received considerable attention for the control of viscosity. However, the existing triggers are mostly temperature [16,17], pH [18,19], light [20,21], and redox [22,23] responses, and these conventional stimuli often have limits or drawbacks, such as severe application circumstances, contamination of the system by by-products and additives, etc. Therefore, there is a pressing need to study a novel stimulus, and simpler and truly green wormlike micelles. As a commonly accessible, inexpensive, non-toxic, biocompatible, and non-combustible gas, CO_2_ has been used in surfactants [24], solvents [25], solutes [26], and polymers [27] due to its superior response qualities. Zhang et al. described a new CO_2_-responsive wormlike micellar system based on natural erucic fatty acid and investigated the rheological properties of its solution [28]. pH-responsive wormlike micelles are micellar systems containing pH-responsive hydrotropes (such as carboxyl or amine groups, etc.) that cause changes in the hydrophilic and lipophilic equilibrium values of surfactant molecules by interacting with H^+^ or OH^−^ ions, or by structural changes of counter ions, leading to the disruption and reconstruction of the wormlike micelle structure and achieving macroscopic changes in viscoelasticity. When the pH of alkylamidopropyl carboxylate betaine-based surfactant was elevated from near 0 to 4.44, Zhao et al. [29] discovered that the energy storage modulus of the solution increased from 20 Pa to 80 Pa, i.e., the elasticity increased, and a further rise led to gel formation. Hassan’s group [30] also found that when pH increased from 2.9 to 12.3, a solution containing cetrimonium bromide/o-aminobenzoic acid changed from a water-like Newtonian fluid to a shear-thinning fluid with a continuously decreasing critical shear rate. The Feng group [31] produced a viscoelastic wormlike micelle with pH-stimulated responsiveness by combining *N*-mustard propyl-*N,N*-dimethyl-tertiary amine and maleic acid (molar ratio, 2:1). When the pH reaches 6.20, the long-chain tertiary amine was protonated to create cationic quaternary ammonium salts; in conjunction with maleic acid, a pseudo-Gemini surfactant structure is generated by electrostatic attraction of non-covalent bonds, self-assembling to form wormlike micelles. When the pH reaches 7.29, the long-chain tertiary amine deprotonates and loses its cationic charge, causing the pseudo-Gemini structure to disappear, and the wormlike micelle network collapses. Consequently, it is anticipated that small-molecule tertiary amines with CO_2_ stimulation in the protonated state and anionic surfactants will also be capable of forming a novel kind of surfactant with a “baryon-like” structure via non-covalent bonding.

The creation of worm micelles by pseudo-Gemini surfactants has been described, but not the formation of worm micelles by pseudo-tetrameric surfactants. Inspired by the CO_2_ stimulation response feature of small-molecule amines, this work focuses on the preparation of pseudo-tetrameric surfactants through non-covalent bonding with conventional anionic surfactants. The feasibility and generalizability of creating an anionic worm-like micellar system with a CO_2_ stimulation response are studied.

## 2. Results and Discussion

### 2.1. CO_2_ Switchable Behavior

**Scheme 1:**
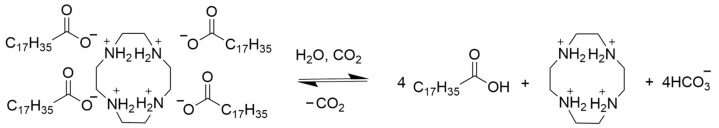
The CO_2_ switching process of the mixed surfactant SA/cyclen.

Our team previously investigated a CO_2_ switchable pseudo-tetrameric surfactant combination produced by mixing oleic acid and four-amine-containing cyclic polyamine (cyclen) [32]. The effect of surfactant hydrophobic tail chain length is examined in this research; the oleic acid is replaced with stearic acid (SA) to avoid the effect of double bonds. The solution of SA/cyclen (300 mM) is produced by combining SA (1200 mM) and cyclen (300 mM) in a precise 4:1 stoichiometric molar ratio (Scheme 1). The as-prepared SA/cyclen solution is viscous; when CO_2_ is continuously injected into the solution, the viscosity of the SA/cyclen solution gradually decreases.

**Figure 1:**
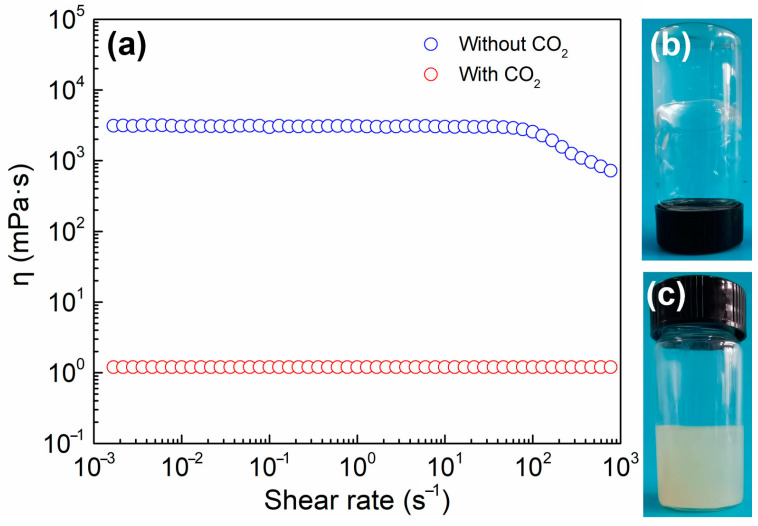
(**a**) Steady rheology of SA/cyclen (300 mM) in the absence or presence of CO_2_ at 25 °C; (**b**) the physical appearance of SA/cyclen (300 mM) in the absence of CO_2_ at 25 °C; (**c**) the physical appearance of SA/cyclen (300 mM) in the presence of CO_2_ at 25 °C.

Figure 1a depicts the viscosity of the SA/cyclen solution in order to explore the distinct flow phenomena of the solution before and after the addition of CO_2_; the appearance of the SA/cyclen solution is shown in Figure 1b,c. After CO_2_ is added, the SA/cyclen solution is a standard Newtonian fluid, such as pure water; its shear viscosity is not changed by the shear rate, and its viscosity is just 1.2 mPa·s; the protonated SA is insoluble in water forming the opaque emulsion visible in Figure 1c. After bubbling N_2_ to eliminate CO_2_, the viscosity of the SA/cyclen solution rises to 3000 mPa·s, as Figure 1b shows, an increase of about 2500 times, and a viscosity plateau is seen at low shear rates (Figure 1). When the shear rate reaches a critical value, the shear viscosity of the solution gradually decreases with the increase of the shear rate. Typically, this shear-thinning behavior is believed to be the result of the rearrangement of the entangled structure formed by wormlike micelles in solution under stress gradually aligning parallel to the direction of the flow field; thus, it is regarded as one of the most compelling types of evidence for the existence of wormlike micelles [7].

**Figure 2:**
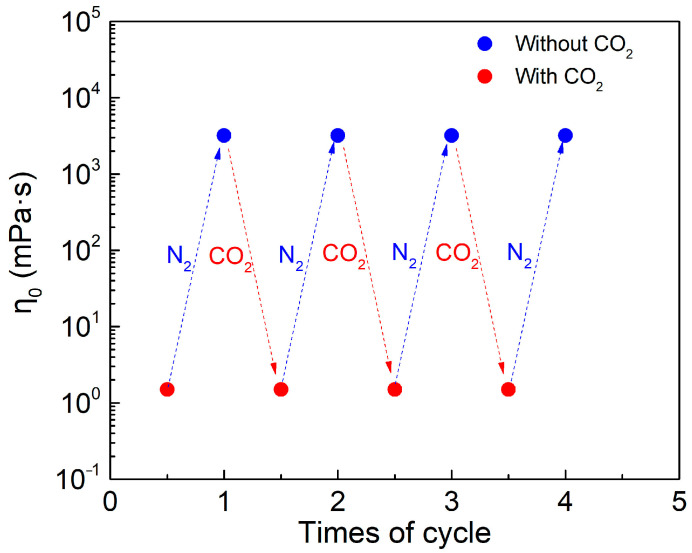
The reversible switchability of SA/cyclen (300 mM) in the absence or presence of CO_2_ at 25 °C; the shear rate is 10 s^−1^.

**Figure 3:**
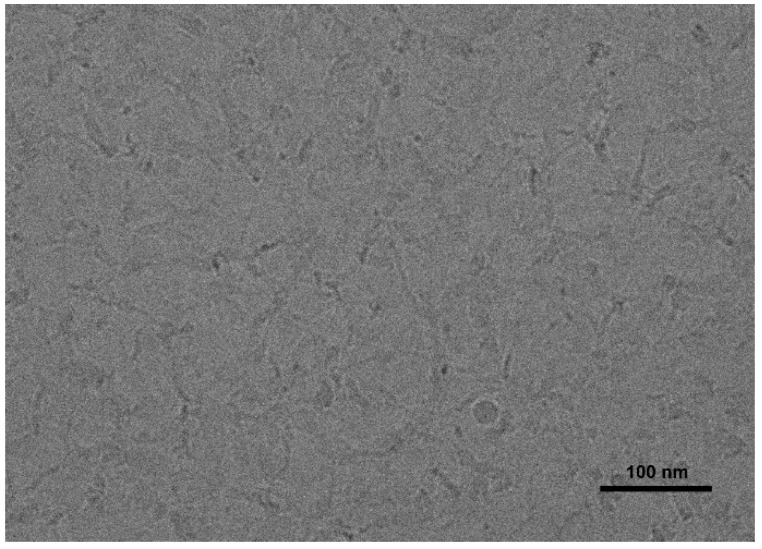
Cryo-TEM image of SA/cyclen (300 mM) in the absence of CO_2_; The scale bar is 100 nm.

Figure 2 shows the switchable states of SA/cyclen solution (300 mM). The pH value of the solution was in the range of 12.2 to 12.4 at the initial stage and in the absence of CO_2_, and it decreased to about 8.3 after the full introduction of CO_2_. The original high viscosity of the solution is restored when the CO_2_ is added into solution. The viscosity remains the same when the introduction and removal of CO_2_ are cycled back and forth four times (Figure 2). Figure 3 shows the microstructure of SA/cyclen (300 mM) in the absence of CO_2_. Before CO_2_ is introduced, many flexible filamentary aggregates a few nanometers in diameter and a few micrometers in length form a three-dimensional mesh structure. After the CO_2_ is discharged, it is assumed that the network structure disappears and is replaced by spherical structures. This comparison shows that the substantial variation in the macroscopic rheological characteristics of SA/cyclen solution (300 mM) with CO_2_ uptake and discharge is attributable to the creation and disruption of an entangled network of wormlike micelles.

### 2.2. Mechanism of CO_2_ Switching

To further investigate the mechanism of CO_2_ switching of SA/Cyclen, we measured the pH of the solutions and investigated whether aqueous solutions of SA and cyclen respectively have CO_2_ switchability. The optimal ratio between SA and cyclen was also investigated.

#### 2.2.1. Testing of pH

**Figure 4:**
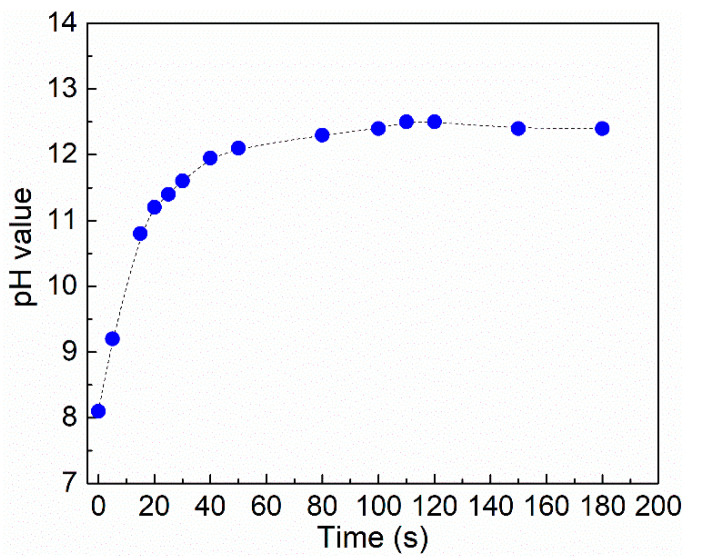
Evolution of pH of SA/cyclen solution (300 mM) with increasing N_2_ bubbling time. (Temperature, 25 °C; flow rate of N_2_, 0.1 L·min^−1^).

To acquire a deeper understanding of the process behind the development and dissolution of the worm-like micellar entanglement network owing to the addition and discharge of CO_2_, the pH changes of the solution were tested (Figure 4). the results demonstrate that, as the bubbling time of N_2_ increases, the pH increases from 8.1 to 12.4, indicating that the removal of CO_2_ causes deprotonation of the SA/cyclen solution (300 mM). Fatty acids with CO_2_ still reversibly produce worm micelles mostly via pH changes and the reversible protonation of the fatty acids [28,33].

#### 2.2.2. Viscosity of Respective Solutions of SA and Cyclen

Although the molecules of SA and cyclen can be altered by the addition of CO_2_, the macroscopic properties of the separate solutions of cyclen or SA (concentration of SA, 1200 mM; concentration of cyclen, 300 mM) did not vary appreciably and always showed Newtonian fluid behavior with low viscosity between 0.8 and 1.5 mPa·s, which is quite different from the combination of SA/cyclen. The mixing of SA and cyclen leads to the formation of pseudo-tetrameric surfactants, which are expected to generate micelles that resemble wormlike micelles, as shown in Figure 3.

#### 2.2.3. Ratio of SA and Cyclen

**Figure 5:**
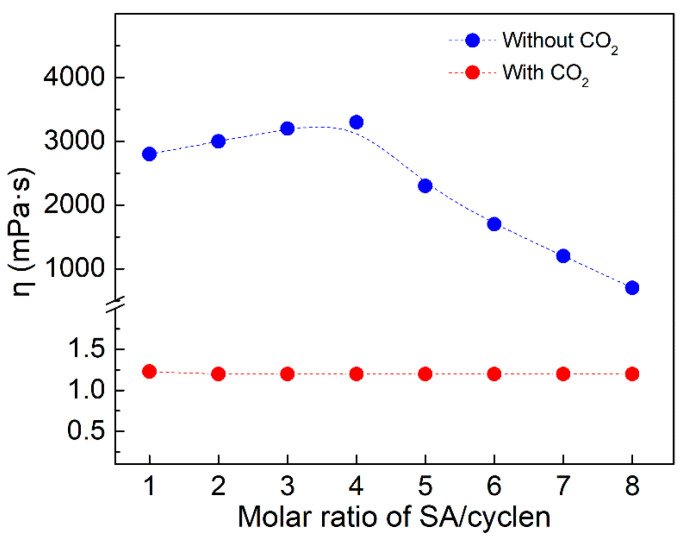
Change in viscosity with varying molar ratio of CSDS/CTMPDA with SA concentration fixed at 1200 mM. (Temperature, 25 °C; shear rate, 10 s^−1^).

Fixing the concentration of SA at 1200 mM and varying the concentration of cyclen (Figure 5), it was found that the viscosity of the mixed solution did not change with varying molar ratio of SA/cyclen in the presence of CO_2_; whereas, after the removal of CO_2_, the viscosity reached 3000 mPas and remained nearly constant up to a ratio of SA/cyclen of about 4. This provides more evidence that the tetrameric surfactant exists and that the viscosity of the SA/cyclen solution is mostly determined by the concentration of the tetrameric surfactant.

Combining the above experimental results, the mechanism of the CO_2_-switchable behavior of SA/cyclen can be summarized as follows: In the presence of CO_2_, mixed SA/cyclen behaves more like pure SA in water, resulting in low viscosity; however, when N_2_ is introduced to discharge CO_2_, SA becomes an anionic surfactant. Under the influence of electrostatic attraction, one molecule of cyclen and four molecules of SA combine to form a pseudo-tetrameric surfactant with non-covalently interaction, which microscopically forms viscoelastic worm-like micelles. When CO_2_ is added, the carboxyl group of SA is protonated, causing electrostatic attraction to decrease, and the structure of the pseudo-tetrameric surfactant collapses, causing the worm-like micelles to disassemble. The cyclen molecule serves as a spacer for the pseudo-tetrameric surfactant. This mechanism is consistent with the previously reported mechanism of the anionic surfactant sodium dodecyl sulfate and diamines switched by CO_2_ [34].

### 2.3. Effect of Sparging Time for CO_2_

**Figure 6:**
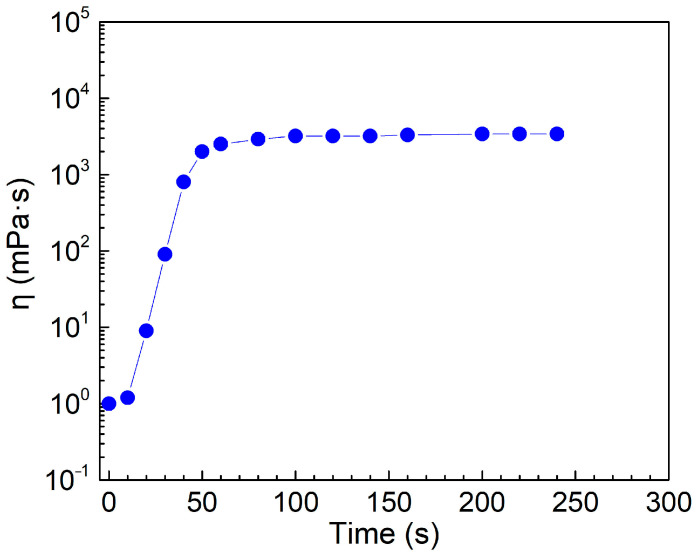
Change in viscosity of SA/cyclen solution (300 mM) with N_2_ bubbling time for the removal of CO_2_ (Temperature, 25 °C; shear rate, 10 s^−1^; flow rate of N_2_, 0.1 L·min^−1^).

Figure 6 illustrates the variation of η in the SA/cyclen solution (300 mM) with saturated CO_2_ with the bubbling time of N_2_ to remove CO_2_. At the commencement of CO_2_ removal, η climbs quickly over a short period, then plateaus after around 100 s, showing a maximum value of the viscosity. This turning point correlates with the variation of pH with the period of bubbling CO_2_, i.e., when η approaches equilibrium, the pH decreases to its lowest. In the presence of CO_2_, SA is protonated, but in the absence of CO_2_, SA becomes an anionic surfactant that combines with cyclen to form a pseudo-tetrameric surfactant with non-covalent bonds, resulting in a rapid rise in η. After approximately 60 s, the change in η tends to moderate, meaning a maximum concentration of the pseudo-tetrameric surfactant has been reached and no new networks of worm-like micelles are formed. This demonstrates that the viscosity of the SA/cyclen combination is significantly influenced by the concentration of the pseudo-tetrameric surfactant.

### 2.4. Effect of SA/Cyclen Concentration

**Figure 7:**
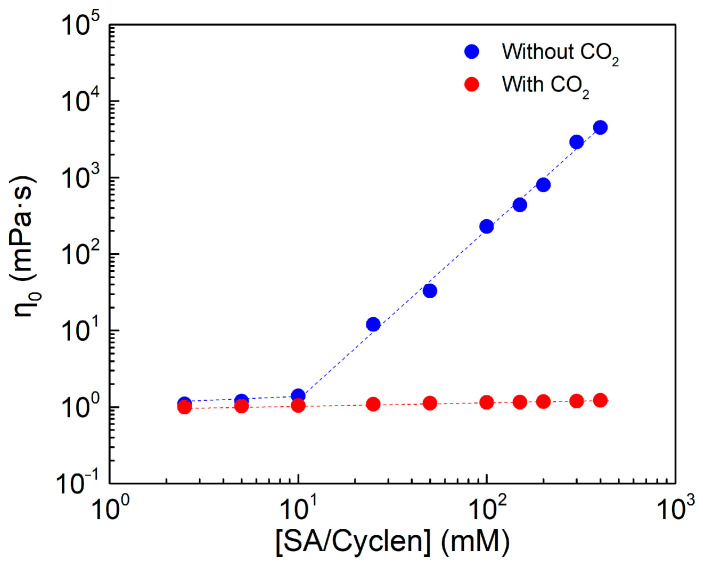
The effect of concentration on viscosity of SA/cyclen solution without and with CO_2_ at 25 °C, the shear rate is 10 s^−1^.

Figure 7 demonstrates that, in the presence of CO_2_, η_0_ increases very slowly with increasing surfactant concentration, and there is no discernible change in the entire concentration range; after the removal of CO_2_, the curve is clearly divided into two distinct regions, and the concentration corresponding to the inflection point is the critical overlapping concentration.

### 2.5. Effect of Amine Types

**Figure 8:**
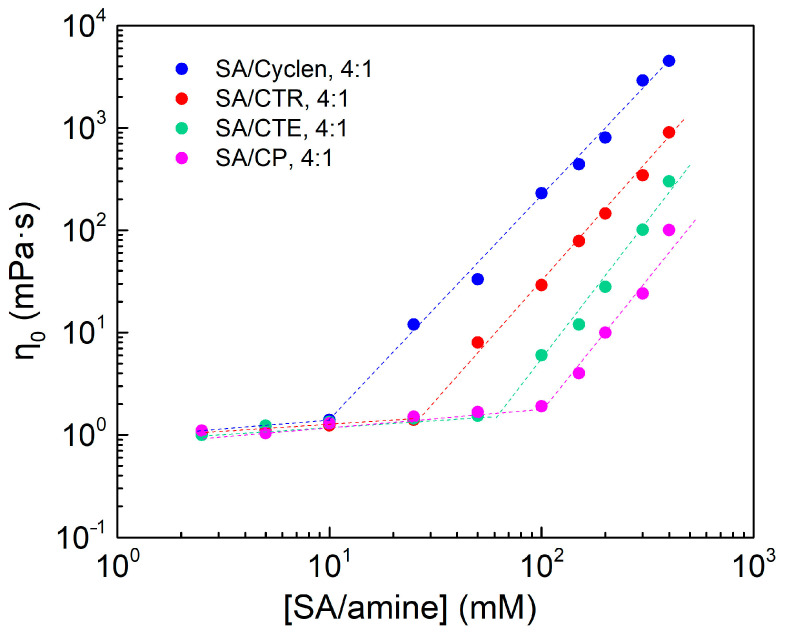
The effect of concentration on viscosity of SA/amine at 25 °C, the shear rate is 10 s^−1^.

The connection between η and concentration for a pseudo-tetrameric surfactant with SA and different types of amines is shown in Table 1 and Figure 8. The curves of viscosity are clearly split into two sections. In the dilute solution zone, η grows slowly with increasing concentration; in the sub-concentrated solution region, η grows exponentially with increasing concentration. Under the same concentration—100 mM for example—η of the SA/cyclen solution has the largest value, indicating that when the spacer consists of two methylene groups, the pseudo-tetrameric surfactant formed has the greatest ability to self-assemble into worm-like micelles. Table 1 and Figure 8 demonstrate that the size of the spacer in the amine molecule is one of the most influential elements influencing the formation of wormlike micelles for pseudo-tetrameric surfactants.

### 2.6. Effect of Hydrophobic Chain Length

**Figure 9:**
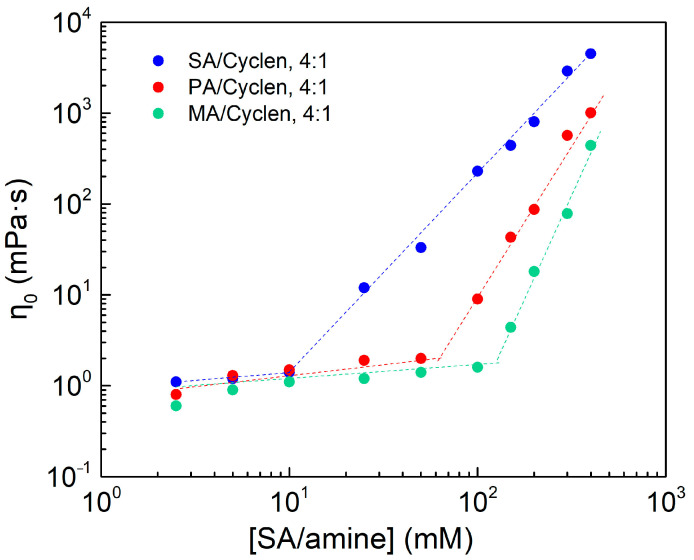
The effect of concentration on viscosity of long-chain carboxylic acid/cyclen at 25 °C, the shear rate is 10 s ^−1^.

Figure 9 shows the three distinct hydrophobic tail chain lengths of tetrameric surfactant micelle solutions may also be separated into slow- and rapid-growth regions. Table 2 shows that the longer the hydrophobic chain, the higher the viscosity; and the reason is that a stronger hydrophobic associative force of the surfactant improves the creation of wormlike micelles. There is a possible relationship between the inflection point and the critical micelle concentration (CMC) of the fatty acids. The CMC of the fatty acids increases by a factor of two to eight for every two carbon atoms removed from the alkyl chain [35]. The pattern of CMC is comparable to that of the inflection point in Figure 9.

## 3. Experimental Procedures

### 3.1. Materials

Stearic acid (SA, 95%), palmitic acid (PA, 99%), myristic acid (MA, 99%), cyclen (97%), 1,4,8,12-tetraazacyclopentadecane (CP, 97%), and 1,4,8,11-tetraazacyclotetradecane (CTE, 98%) were purchased from Sigma-Aldrich (Burlington, MA, USA). 1,4,7,10-tetraazacyclotridecane (CTR, 95%) was supplied by BidePharm Co. (Shanghai, China). These reagents are used directly after purchase, without further purification. CO_2_ (≥99.998%) and N_2_ (99.998%) were supplied by Xuyuan Chemical Industry Co. Ltd. (Chengdu, China) and were used as received. Ultrapure water (resistivity, 18.25 MΩ·cm^−1^) was obtained using an ultrapure water purification system (Chengdu Ultrapure Technology Co., Ltd., Chengdu, China).

### 3.2. Preparation of Solution

Unless otherwise specified, all samples were produced in a 4:1 molar ratio of fatty acid to amine. The fatty acid and amine were accurately weighed, dissolved in ultrapure water, and placed in an oscillating water bath (VWR Sheldon 1217, VWR, Part of Avantor, Radnor, PA, USA) at a constant temperature for at least 24 h to obtain a homogenous clarified solution of the mother liquor. The temperature range of the oscillating water bath was from 5 to 99.9 °C; its temperature sensitivity was ±0.07 °C. A typical example is SA/cyclen as the combination of fatty acid and amine. Concentrations were computed as “SA/cyclen”, i.e., the combination of 4 mol·L^−1^ of SA and 1 mol·L^−1^ of cyclen are recorded as 1 mol·L^−1^ of SA/cyclen. The mother liquor was diluted with ultrapure water to produce the other solutions. The other solutions were produced by diluting the mother liquor with ultrapure water. Then, CO_2_ was bubbled into the samples at a flow rate of 0.1 L·min^−1^ while oscillating continuously. Unless otherwise specified, the CO_2_ was fed through until the pH of the system stopped changing. To remove CO_2_ from the system, N_2_ was supplied into the surfactant solution at a flow rate of 0.1 L·min^−1^ until the pH remained stable. Prior to any measurements, all samples were kept in a water bath at the measurement temperature for at least 24 h.

### 3.3. pH Testing

The reagent bottle holding the sample to be tested was put in a water bath maintained at a constant temperature. After 30 min, the electrode of a pH meter (PB-10 basic, Sartorius AG, Gottingen, Germany) was submerged in the sample solution until the temperature and pH readings reached equilibrium. Each sample was tested three times in parallel with a relative variation of less than 2%, with the average of the three readings being taken as the pH value of the sample. Before each measurement, the pH meter was calibrated using a standard buffer solution.

### 3.4. Rheological Performance

The rheological qualities were investigated using a Physica MCR301 rheometer (Anton Paar, Graz, Austria) with a 13.33 mm-radius concentric-axis cylinder CC27 rotor and a rotor (radius, 14.46 mm). The sample temperature was monitored with an accuracy of 0.01 °C utilizing a Peltier thermocouple temperature control device in the rheometer. On the measuring equipment, a solvent trap was used to limit sample evaporation. Each time the instrument was turned on, it was calibrated using Cannon standard oil to decrease measurement mistakes. To ensure that the samples were in equilibrium, they were placed in a water bath at the measuring temperature for more than one hour before being measured and then placed in a revolving cylinder for five minutes. The steady-state shear experiments were performed in rate-controlled mode with shear rates ranging from 10^−3^ to 10^2^ s^−1^.

### 3.5. Cryo-TEM

The microstructure of the micellar solutions was observed by Cryo-TEM (JEM2010, JEOL Ltd., Tokyo, Japan). The working and instrumental conditions were: a working accelerating voltage of 200 kV, the image acquisition system was a CCD imaging system with a Gatan 832 camera, the controlled temperature of the cryo-transfer frame Gatan-626 was not higher than −174 °C, and the microsieve was a Quantifoil 1.2/1.3. The thoroughly dissolved micellar solution was kept stable in a water bath at the set temperature for 1 h, then 3 μL of the sample was quickly pipetted onto the carbon grid. The resulting thin layer of micellar solution applied uniformly to the carbon grid was then quickly immersed in liquid ethane cooled to −175 °C by liquid nitrogen for quenching and cooling, and the treated sample was then stored in liquid nitrogen for Cryo-TEM observation.

## 4. Conclusions

Electrostatic attraction bonds the quaternary ammonium salt of cyclen to four molecules of SA, generating a pseudo-tetrameric surfactant, increasing the volume of the hydrophobic tail group, and forming a viscoelastic wormlike micelle. After the introduction of CO_2_, the pseudo-tetrameric surfactant is destroyed and reverted to protonated SA, leading to low viscosity. The enhanced viscosity switched by CO_2_ stimulation was stable across several cycles in testing. The impacts of several factors on the rheological characteristics of the wormlike micellar structure were analyzed independently, including the introduction time of CO_2_, surfactant concentration, size of the hydrophobic tail group, and the type of amines. In addition, the pseudo-tetrameric surfactant is generated using only commercially available, affordable anionic surfactants and cyclen in a 4:1 molar ratio in aqueous solution to create CO_2_-switchable wormlike micelles without complicated chemical synthesis. Moreover, the length of hydrophobic tail chains for SA and the size of spacer groups for cyclen also influence the formation of wormlike micelles, rather than the concentration of the surfactant. A longer hydrophobic chain of the surfactant and shorter spacer group of the amines improve the formation of worm-like micelles.

This approach has numerous benefits, including a single component and ease of preparation. The wormlike micelles described in this paper are more accessible and environmentally friendly than other CO_2_-switchable micelles because they can be created by merely adjusting the pH of a solution containing a natural fatty acid and an amine, without the requirement for sophisticated organic synthesis or the addition of hydrotropes. It is anticipated that this pseudo-tetrameric surfactant can be employed as a clean fracturing fluid in oil and gas field production enhancement activities; for example, if a dry well cannot be automatically broken, this can be remedied by eliminating CO_2_ from the surfactant. Moreover, results reveal that this pseudo-tetrameric surfactant was sensitive to CO_2_ and had potential channel-blocking performance in porous media, which would be efficient for enhanced oil recovery in diverse deposits. Thus, this pseudo-tetrameric surfactant could serve as inspiration for field applications of CO_2_-sensitive gel technology.

## Figures and Tables

**Table 1:** Comparison of the viscosity of SA mixed with different amines (molar ratio, 4:1).

Amines	IUPAC Name	Molecular Structure	Number of Carbon Atoms of Spacer Group	Viscosity ^a^ at 100 mM (mPa·s)
Cyclen	1,4,7,10-tetraazacyclododecane	_ 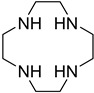 _	2, 2, 2, 2	231
CTR	1,4,7,10-tetraazacyclotridecane	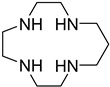	3, 2, 2, 2	29.2
CTE	1,4,8,11-tetraazacyclotetradecane	_ 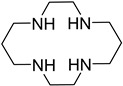 _	3, 2, 3, 2	6.1
CP	1,4,8,12-tetraazacyclopentadecane	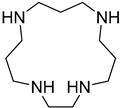	3, 3, 3, 2	1.9

^a^ Condition: Temperature, 25 °C; shear rate, 10 s^−1^; concentration of SA, 400 mM; concentration of amine, 100 mM.

**Table 2:** Comparison of different long-chain carboxylic acids mixed with cyclen (molar ratio, 4:1).

Long-Chain Carboxylic Acid	Reagent Name	Number of Carbon Atoms Contained in Hydrophobic Chain	Viscosity ^a^ at 100 mM (mPa·s)
SA	Stearic acid	18	231
PA	Palmitic acid	16	8.9
MA	Myristic acid	14	1.6

^a^ Condition: Temperature, 25 °C; shear rate, 10 s^−1^; concentration of carboxylic acid, 400 mM; concentration of cyclen, 100 mM.

## Data Availability

The data presented in this article will be available upon request.
